# Individual Behavioral Reactions in the Context of Food Sensitivities in Children with Attention-Deficit/Hyperactivity Disorder before and after an Oligoantigenic Diet

**DOI:** 10.3390/nu13082598

**Published:** 2021-07-28

**Authors:** Elena Yorgidis, Lisa Beiner, Nicola Blazynski, Katja Schneider-Momm, Hans-Willi Clement, Reinhold Rauh, Eberhard Schulz, Christina Clement, Christian Fleischhaker

**Affiliations:** Department of Child and Adolescent Psychiatry, Psychotherapy and Psychosomatics, Faculty of Medicine, Medical Center—University of Freiburg, D-79104 Freiburg, Germany; elena.yorgidis@gmx.de (E.Y.); lisa.beiner@uniklinik-freiburg.de (L.B.); n.blazynski@gmail.com (N.B.); katja.schneider-momm@uniklinik-freiburg.de (K.S.-M.); hans-willi.clement@uniklinik-freiburg.de (H.-W.C.); reinhold.rauh@uniklinik-freiburg.de (R.R.); profeberhardschulz@gmx.de (E.S.)

**Keywords:** adolescents, attention-deficit/hyperactivity disorder, behavior, child, diet, elimination, food intolerance, nutrition, Oligoantigenic Diet

## Abstract

The influence of food intake on behavior problems of children with Attention-Deficit/Hyperactivity Disorder (ADHD) was already described in the early 20th century. Eliminating food components by using the Oligoantigenic Diet (OD) leads to reduction of ADHD symptoms for more than two-thirds of patients. The aim of our study was to reveal how to identify foods having an impact on ADHD symptomatology. Therefore, 28 children with ADHD participating in this uncontrolled, open trial were examined before and after a restricted elimination diet. They kept a daily 24-h recall nutrition and behavior journal and filled out the abbreviated Conners’ scale (ACS) to identify foods which increased ADHD symptoms. The study was completed by 16 children (13 m/3 f). After four weeks of elimination diet the individual food sensitivities were identified in a reintroduction phase. A repetitive increase of ADHD symptoms by at least two points in ACS after food introduction hints at food sensitivity. Twenty-seven food sensitivity reactions were identified. Most of the participants were sensitive to more than one food. Food intolerances could not be identified without preceding OD. The combination of OD and subsequent food challenge appears as a valid method to identify individual food sensitivity in ADHD.

## 1. Introduction

With a worldwide prevalence of 5.3% among children and adolescents, Attention-Deficit/Hyperactivity Disorder (ADHD) is the most common behavioral disorder [1,2]. ADHD occurs across cultures in about 5% of children and about 2.5% of adults. In children there is a gender related ratio of 2:1 (male: female), [3]. The mechanisms triggering ADHD have not yet been fully identified. Genetic predisposition and pre-, peri- and postnatal environmental influences play a decisive role as do multiple interacting factors [4,5,6]. Nutrition also plays a role in the development of ADHD [7]. Previous study results have supported the theory that ADHD is an expression of a genetically determined neurodevelopmental disorder. Depending on the degree of severity, the guidelines recommend different treatment options, including parent training, behavioral therapy, pharmacotherapy, and dietary interventions [8,9,10].

As early as 1922, Shannon noted an increase in restlessness and sleep disorder in children in association with food allergies. After eliminating foods such as tomatoes, eggs and grains, there was reduction or even disappearance of ADHD symptoms [10]. In 1983, Egger et al. were the first to carry out the Oligoantigenic Diet (OD)—as a dietary diagnostic method to identify food allergies in the field of allergology—in the context of ADHD. Foodstuffs during diet were consciously reduced to very few hypoallergenic foods. The choice of food was initially kept to a minimum. Approved foods were hypoallergenic, mainly including foods which rarely caused adverse reactions. Throughout the OD the ADHD patients showed significant improvements: of 76 participants in this study, 62 children reduced their symptoms. Furthermore, 21 patients no longer met diagnostic criteria for ADHD. Most children responded to two to seven different foods. Re-exposure to the foods caused reappearance or intensified symptoms of ADHD [11]. A study by Pelsser et al. (2009) also showed a reduction in ADHD symptoms after an OD. Here 60% of the participants showed a reduction in symptoms of at least 50% measured with the ADHD Rating Scale [12].

Since then, further studies have shown that nutrition is a strong mediator and/or moderator of ADHD symptoms [8,9,10,11,12,13,14,15,16,17,18,19,20,21,22]. Dietary interventions in ADHD including elimination diets have shown highly significant effects [13,14,15,17,18,19,20,21,22] with effect sizes up to Cohen’s *d* = 5.0 in unblinded studies [7].

Severe dietary interventions such as restricted elimination diets do have a clear impact on daily life and therefore cannot be kept under blinded conditions. In order to minimize the various biases which influence the assessment of ADHD symptoms, Dölp et al. [22] used blinded video ratings to evaluate their primary outcome diagnostic tool, ARS, in the context of dietary intervention. The results showed hardly any difference between blinded and unblinded ratings. Dölp et al. found that OD can lead to symptom reductions in food sensitive children and adolescents with ADHD. After 4 weeks of diet, approximately 60% of the patients showed significant improvement in their condition in ARS [22].

The objective of the present study is to answer the following questions: is it possible to identify foods that intensify typical ADHD symptoms in children by applying OD? Do the participants show different reactions to the same food? Can individual food sensitivities already be identified in a pre-diet phase? Can strong leads to later diagnosed food sensitivities be seen already in the anamnesis?

## 2. Materials and Methods

The study was approved by the Ethics Committee of Freiburg (application number 111/14) in accordance with the World Medical Association’s Declaration of Helsinki. Patients and parents gave written informed consent before participating in the study.

### 2.1. Participants

The study took place at the Department of Child and Adolescent Psychiatry, Psychotherapy and Psychosomatics of the Medical Center, University of Freiburg. Psychotherapists and general practitioners informed their patients about the study. They were then recruited by study staff. Some participants also became aware of the study via the local press or information on the Internet. Families did not receive any reward for their collaboration in the study.

Interested participants were instructed in detail on the procedure of the study, either in group meetings or individually. ADHD diagnoses were confirmed with Kiddie-SADS-Present and Lifetime Version (K-SADS-PL) [23]. Table 1 shows the characteristics of the participants.

Four of the participants (25%) had a disease at the beginning of the study (rhinitis (*n* = 2), influenza (*n* = 1), gastroenteritis (*n* = 1)).

### 2.2. Inclusion and Exclusion Criteria

Children and adolescents between the age of 7 and 18 attending at least 2nd grade of a general education school with a confirmed ADHD diagnosis according to the criteria of ICD-10 were included in the study. Children and their parents had to sign for informed consent.

Exclusion criteria were severe concomitant disease, neurological or organic comorbidities which cannot be subjected to dietary intervention. Patients could not participate if there was a lack of compliance either of the parents or children, or a lack of reading or writing skills. Concurrent drug therapy of ADHD or participation in other studies at the same time was not allowed. Children were not to be included when following a special diet (e.g., vegetarian, vegan).

### 2.3. Measures

The primary outcome was measured using the ADHD rating scale IV (ARS) that is frequently used in ADHD trials [24,25,26,27,28,29,30,31,32,33,34].

### 2.4. Anamnesis Concerning Food Sensitivity and Allergies in the Beginning

Participants were asked about existing food intolerances, such as allergic reactions to foodstuff or diagnosed food intolerances such as malabsorption or enzyme deficiency.

In the special dietary anamnesis, the children were asked about their preferences and aversions to certain foods.

### 2.5. Conners’ Rating Scale

To assess daily changes of behavior more precisely, the abbreviated Conners´ scale (ACS) was used on a daily basis [15,35,36]. In addition to diagnostics, the Conners´ scales are important for treatment planning, monitoring, and therapy evaluation. The ACS used here is derived from the long version (105 items), the Conners 3^®^. This is composed of the items that achieved the highest scores in patients with ADHD and which react very sensitively to therapy effects. Therefore, they are suitable as a short-term follow-up and therapy evaluation [35]. It consists of ten selected items. The ten items are: (1) restless and overactive; (2) excitable impulsive; (3) disturbs other children; (4) fails to finish things—short attention span; (5) constantly fidgeting; (6) inattentive, easily distracted; (7) demands must be met immediately—easily frustrated, (8) cries often and easily; (9) mood changes quickly and drastically (10) temper outbursts, explosive and unpredictable behavior.

Each item was assessed on a 4-point rating scale which resulted in a total score ranging from 0 to 3. With a time requirement of about five minutes, the ACS is a suitable instrument for daily progress monitoring or therapy evaluation. [36].

### 2.6. Nutrition and Behavior Diary

The nutrition and behavior diary, developed for this study, is based on the allergy diary described by Körner and Schareina [35]. It was kept daily as a 24-h recall protocol by the parents and/or children during the entire study in order to be able to track a temporal and causal relationship between the foods consumed and the occurring ADHD symptoms [34].

Leisure activities that may also affect ACS are also reported e.g., birthday parties, physical training, circus events, and school hiking days.

### 2.7. Procedure

**Figure 1:**
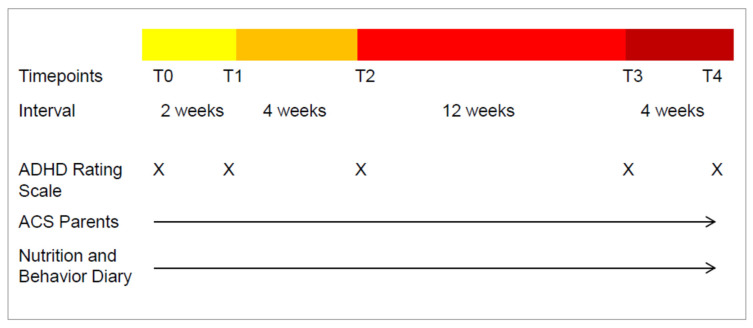
Timescales and measures for each appointment. Yellow bar: pre-diet phase, no change in everyday food intake; Orange bar: diet phase OD; Red bars: food challenge phase, testing the different main food groups. T0: physical examination, Baseline ARS; T1: physical examination, ARS; T2: physical examination, ARS; T3: physical examination, ARS; T4: physical examination, ARS, individual dietary recommendations.

Initial examination, verifying the ADHD diagnosis, and an assessment of medical health status was followed by a one-week retrospective ADHD rating scale IV [24]. T0 was considered baseline. Figure 1 shows the timeline of the study.

Between T0 and T4, parent ACS and 24-h nutrition and behavior protocols were kept daily. Participants were asked to keep their daily eating habits.

From T1–T2 the children and their families are in a 4-week period of OD. During the diet phase, only a limited selection of hypoallergenic food was allowed to be eaten. The structure of this diet was based on the protocol of Egger et al. and Pelsser et al. [11,14]. Supplementation of vitamins and minerals was advised.

At all time-points, physical examination was kept by a medical professional. At T0, the physical condition and basic neurological findings were additionally recorded.

Throughout the study the families were advised by a nutritionist in order to avoid the risk of malnutrition and to facilitate the implementation of the diet.

All children with an improvement of at least 40% in the ARS total between T1 and T2 were considered to be responders [14].

During T2–T4 the reintroduction of usually consumed foods was proceeded at a time interval of 3–4 days testing each food. ACS questionnaire was completed daily and the nutrition and behavior diary was maintained.

Food sensitivities were defined for foods that showed a repetitive increase in symptoms of at least 2 points on the ACS scale after ingestion compared to the three days before baseline. After identifying the intolerant food in the reintroduction phase, it was assessed whether the intolerant food was consumed before the diet.

### 2.8. Statistics

The data of 16 participants (out of 28) that completed the whole study was included in statistical analysis.

In cases in which subsamples exceeded five observations, *t*-tests were performed to compare ACS values on the day of reintroduction (dE0), and one, two or three days after reintroduction (dE + 1, dE + 2, dE + 3, etc.) of a particular food to the values one day before reintroduction (dE − 1). All subsamples were too small to apply more complex statistical analyses such as ANOVA with repeated measurements. All statistical analyses were performed with SPSS version 23.0 (IBM Corporation, Armonk, NY, USA).

## 3. Results

### 3.1. Participants

16 out of 28 children and adolescents completed the study. The proportion by gender corresponds to the general prevalence of ADHD worldwide. We had 81% male and 19% female participants in our study.

During the diet phase 12 participants either dropped out (*n* = 2) of the study at their own request or were considered non-responders (*n* = 10). None of the participants had medical treatment for ADHD during the whole study (Table 2).

### 3.2. ADHD Symptoms According to ARS

The results of this study show that, under careful supervision, children can maintain a 4 weeks OD as documented in the nutrition and health diary. Reductions in ADHD symptoms of 40% or more were seen in 17 participants.

**Figure 2:**
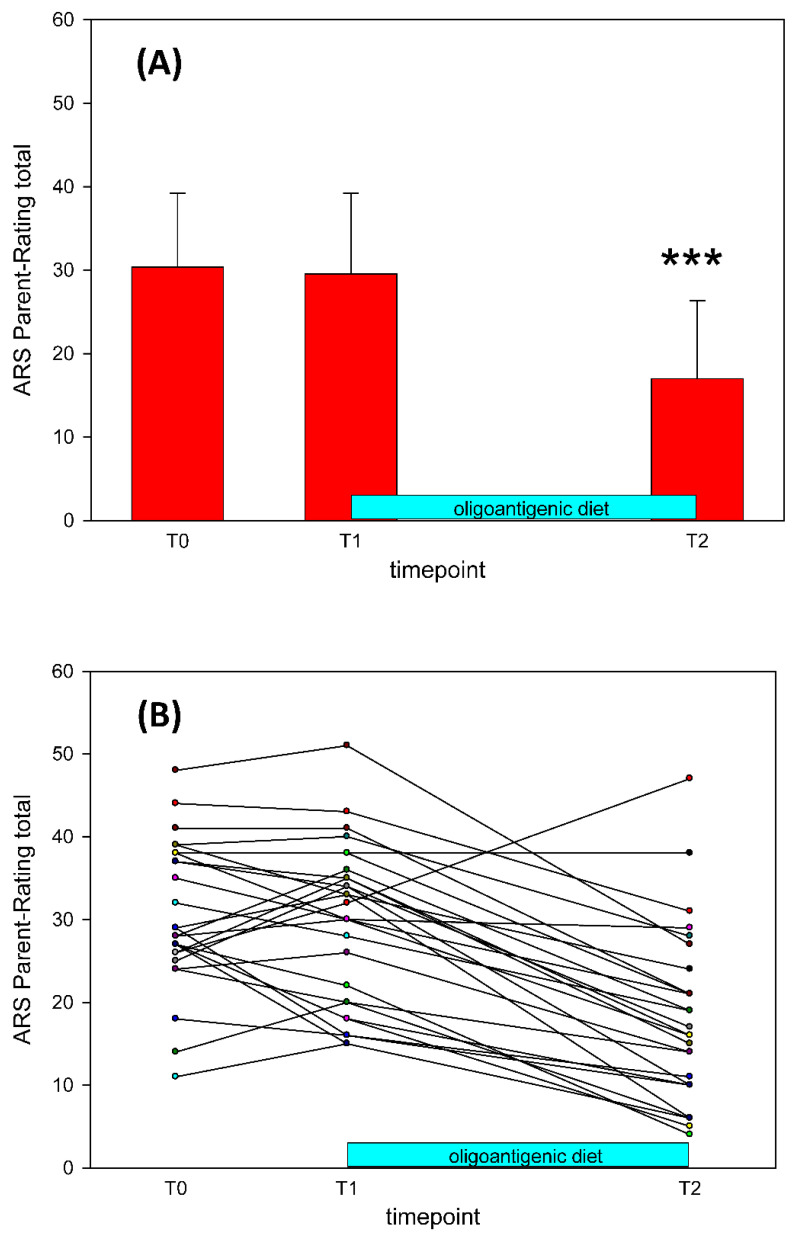
(**A**) Average changes in all participants (*n* = 28) of the ARS scores at each time point (means ± SD), *p* < 0.001 *** (ANOVA with repeated measurements). (**B**) ADHD Rating Scale total scale for all participants in the study. *n =* 28, T0: Baseline, T1: Start of the OD, T2: End of the OD.

At T0, the 28 ARS parent ratings yielded M = 30.36 and SD = 8.87. Figure 2 shows the individual ARS total score trajectories from T0 to T2.

After 2 weeks (T1) of continuing usual nutrition behavior, parental ARS was not significantly different to T0 (T0: M = 30.36, SD = 8.87; T1: M = 29.54, SD = 9.64; *F*(1, 27) < 1).

From the 28 participants starting the diet, 26 (91.6%) completed. As shown in Figure 2A, after 4 weeks of OD (T2) we observed a significant reduction of ARS total score (T2: M = 15.62, SD = 8.05, *F*(1, 25) = 112.34, *p* < 0.0001). The percentage of improvement observed after the diet, according to the change in ARS total score, was 47.4% on average, ranging from 3.3% to 81.8%. Nine children showed at least 40% symptom reductions in both ARS subscales of inattention and hyperactivity/impulsivity. Thirteen children showed at least a 40% reduction in one subscale. Only three of the participants did not respond in either subscale.

According to Storebø et al. [27] a change of 6.6 points on the ARS is considered as the minimum for a clinically relevant difference. 22 of the 26 participants (84%) showed improvements of between 9 and 27 points between T1 and T2. Three of the 26 participants (11%) showed improvements of between 9 and 13 points between T0 and T1.

### 3.3. Identified Food Sensitivity

**Figure 3:**
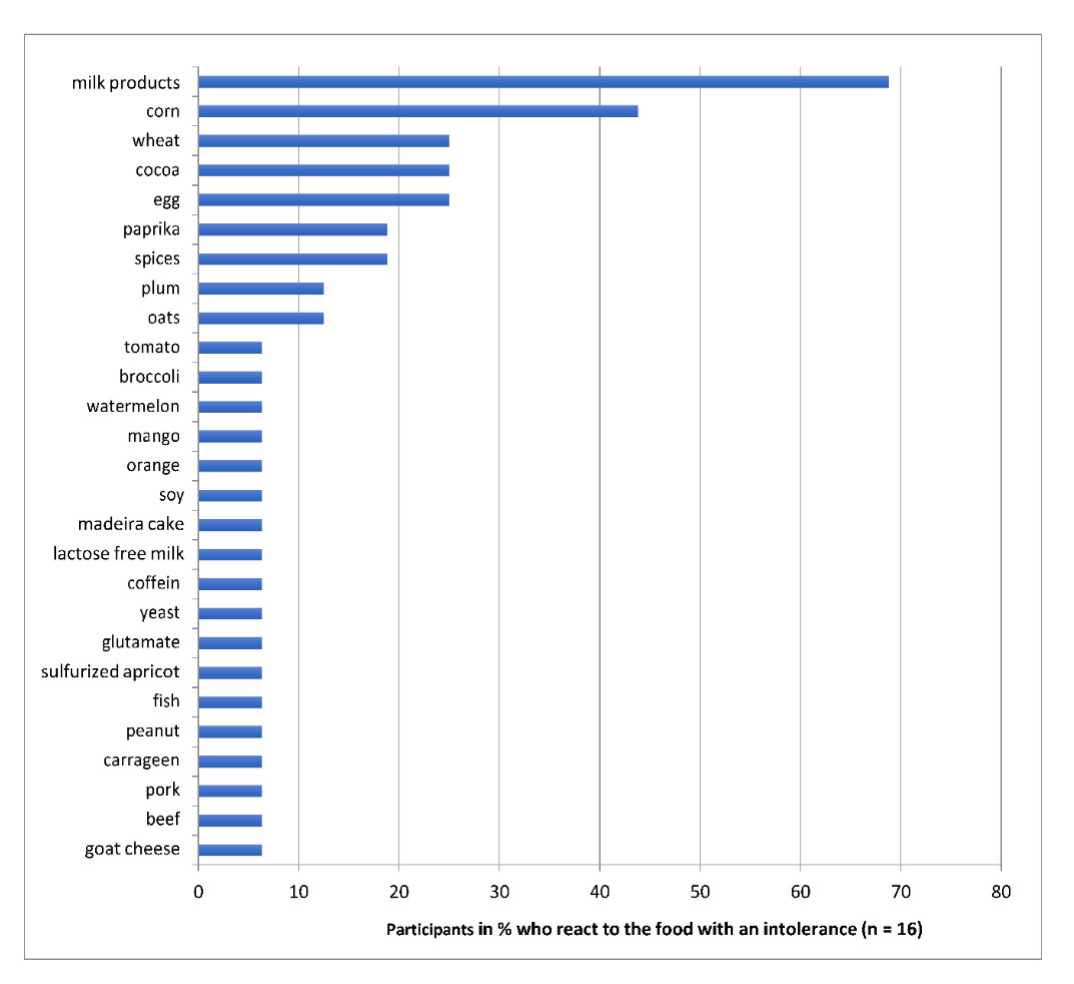
Identified food sensitivities during the study: Percentages of children (*n =* 16) reacting to the respective food with an increase in ADHD symptoms.

The statistical analysis of the reintroduction phase showed 27 different types of food sensitivity (see Figure 3).

### 3.4. Reactions to Intolerant Food after the OD

Each responder to the OD showed individual food sensitivity, reacting to between 1–10 different foods in the food challenge phase. The three most common detected sensitivities are listed below as examples.

#### 3.4.1. Example 1: Milk Products

The group of dairy consisted of milk, yoghurt, curd cheese, cream, cheese and butter made from cow’s milk. Lactose-free products and products made from sheep’s or goat’s milk were not included.

A total of 68.8% (*n =* 11) of respondents showed a change in ACS after taking dairy products. Among the foods tested, milk products are the ones that most often lead to an increase in ACS levels.

On average, the analysis shows a significant increase in ACS on days dE + 1, dE + 2 and dE + 3 with a sustained effect observed for three days.

**Figure 4:**
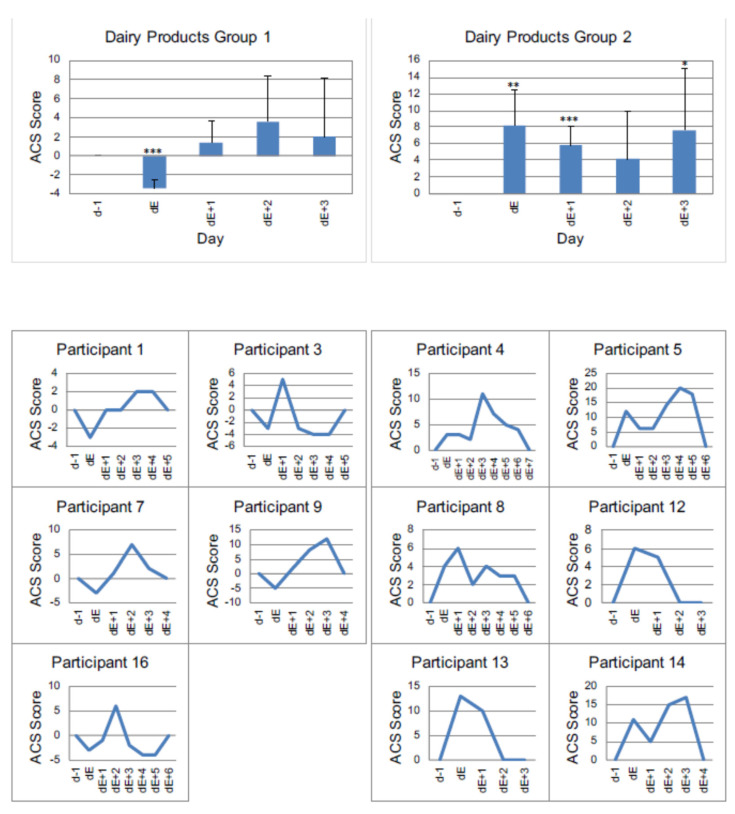
Individual reactions of participants to dairy products, measured by ACS. Group 1 (participants with a decrease of value in ACS at day of reintroduction) vs. Group 2 (participants with an increase of value in ACS at the day of reintroduction) *p* < 0.05 *, *p* < 0.01 **, *p* < 0.001 *** (one sided *t*-test). dE − 1: day before reintroduction, dE: day of reintroduction, dE + 1: day 1 after reintroduction etc.

Evaluating the milk products, we found two differently reacting groups as shown in Figure 4. Group 1 (*n =* 5) initially responded to the intake of milk products with a decrease in ACS followed by a significant increase in the ACS rating after 1 day. In contrast, group 2 (*n =* 6) showed a significant increase in the ACS rating on the day of consumption of dairy.

Figure 4, Table 3 and Table 4 show the daily courses of the mean values as well as the standard deviations of all children in the respective groups. In addition, the individual time courses of each child are displayed.

#### 3.4.2. Example 2: Corn

**Figure 5:**
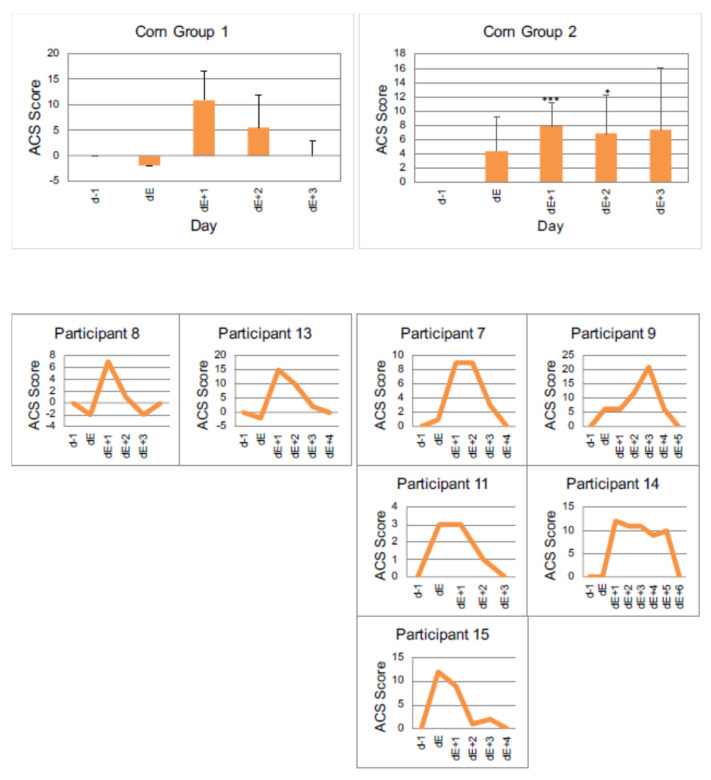
Individual reactions of participants to corn, measured by ACS. Group 1 (participants with a decrease of value in ACS on the day of reintroduction), vs. Group 2 (participants with an increase of value in ACS at the day of reintroduction) *p* < 0.05 *, *p* < 0.001 *** (one-sided *t*-test). dE − 1: day before reintroduction, dE: day of reintroduction, dE + 1: day 1 after reintroduction etc.

Figure 5, Table 5 and Table 6 show the behavioral reactions to corn.

In total, seven children (43.8%) responded to the intake of products containing corn, as shown in Figure 5.

Group 1 shows a decrease in the ACS value on the day of corn introduction, the largest increase (11 points) on day dE + 1 and a value of 0 on day dE + 3.

Group 2 shows an ACS value increase directly on the day the corn is taken. This is significant on day dE + 1 and dE + 2.

#### 3.4.3. Example 3: Grain

**Figure 6:**
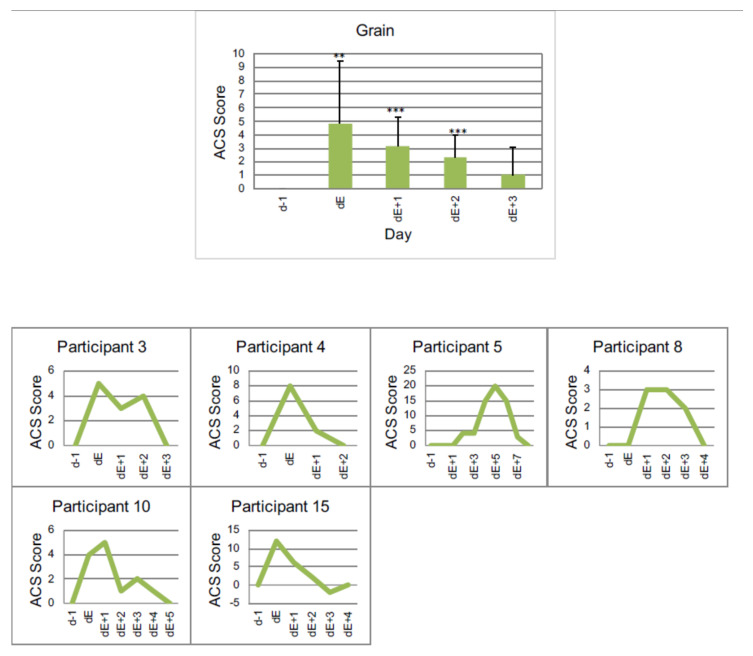
Individual reactions of participants to grain (including oats, wheat and other grain containing gluten) measured by ACS, *p* < 0.01 **, *p* < 0.001 *** (one-sided *t*-test). dE − 1: day before reintroduction, dE: day of reintroduction, dE + 1: day 1 after reintroduction etc.

Figure 6, and Table 7 show the intolerance reaction to grain.

For a total of six children (37.5%) the symptoms worsen when eating grain. These include oats, wheat and other grains containing gluten. Corn was excluded.

### 3.5. Behavioral Reactions to Intolerant Foods before Starting the OD

In the pre-diet phase, 13 out of 16 children consumed several of the observed sensitive foods at the same time. Because of the overlap with the incompatible foods, a reliable prognosis is difficult. In three subjects incompatibilities were observed individually and without overlapping with one another. This was due to the fact that they only reacted to one incompatibility each.

**Figure 7:**
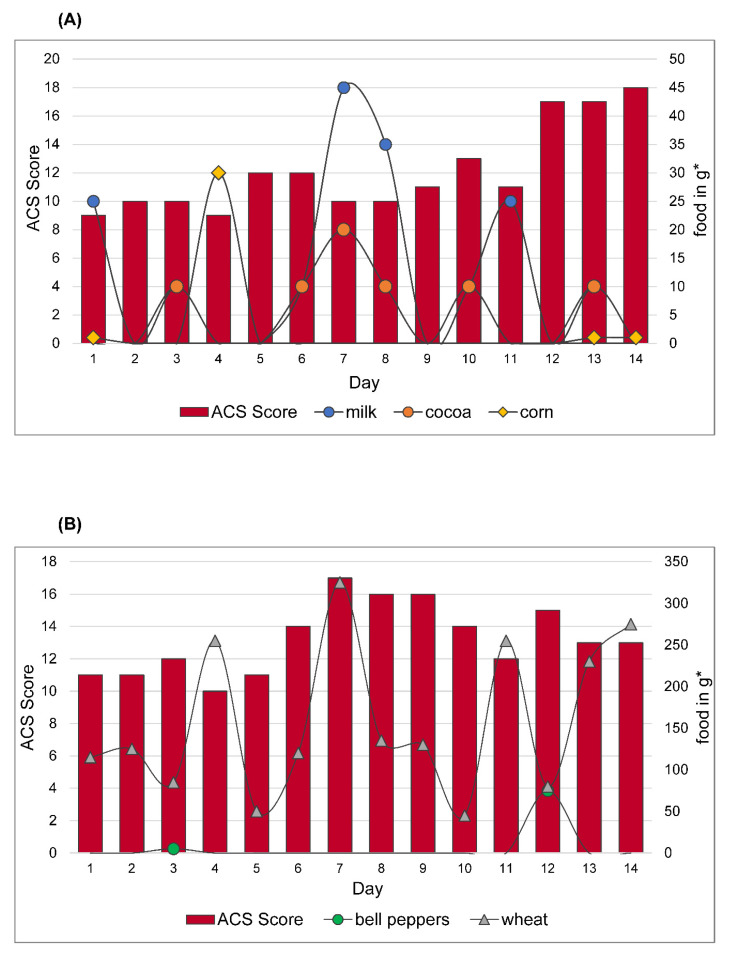
(**A**) Food sensitivity of participant 1. Reactions to different foods in the pre-diet phase. Food sensitivities safely detected during the reintroduction phase: milk, cocoa, peanut and corn. Peanuts are not mentioned because they were not eaten in the pre-diet phase. (**B**) Food sensitivity of participant 2. Reactions to different foods in the pre-diet phase. Food sensitivities safely detected during the reintroduction phase: bell peppers and wheat. ACS value declared by parents, displayed in red bars on the left axis. Amount of intolerable food consumed in grams * shown in lines on the right axis. * estimated amount.

One child showed an increase and decrease in ACS according to the course of the week, so that ACS increased at the weekend without a change in diet (Figure 7A). Overall, none of the children’s behavior disturbances can be attributed to a clear food sensitive reaction just on the basis of the data from the pre-diet phase. Two illustrative examples are shown in Figure 7A,B. Figure 7A shows the food sensitivity of one subject before starting the diet. Food sensitivity was safely detected during the reintroduction phase: to milk, cocoa, peanut and corn. Out of four intolerant foods, three were consumed at different timepoints in the pre-diet phase as shown below.

Looking at the individual foods, milk shows an average decrease in ACS within the first 24 h after consuming by −1.67. In the following days there is an increase in ACS compared to the value before the intake (<24 h = −0.67; 24–48 h = +0.67; 48–72 h= +1).

When cocoa is consumed, an immediate increase in ACS is only notable on day 10 (+2). On day 13 there is a delayed increase the next day (+1). The remaining days (day 3, day 6–8) are inconspicuous, with an average increase of 0.5 in the first 24 h (<24 h = 0.5; 24–48 h = −0.5; 48–72 h= −0.5).

Corn was consumed only in small amounts. On day 4, most corn was consumed in the form of popcorn, with a delayed increase of ACS the next day (+3). (<24 h = −0.67; 24–48 h = +1; 48–72 h= −2).

Due to the weak fluctuations, no clear statement is possible. Overall, possible changes in ADHD symptoms due to intolerant foods overlap, which makes a reliable prognosis of individual food sensitivity almost impossible.

Figure 7B shows the reactions of another participant. Food sensitivities safely detected during the reintroduction phase were to bell pepper and wheat. Wheat was supplied daily in the form of baked goods (bread). On average, there was a mean decrease in ACS in the days after ingestion compared to the initial value (<24 h = −0.75; 24–48 h = 0; 48–72 h= −0.75).

Bell peppers were eaten as spice powder (day 3) or raw (day 12). There is a small increase in ACS (+2) on the respective days, but day 12, when raw paprika was eaten, is particularly noteworthy with an increase from +3 to 15 (<24 h = +2; 24–48 h = 0; 48–72 h= +0.5).

It is also noticeable that on days with physical activity (soccer training, swimming training) ACS is relatively low (day 1, 2, 5) or decreased compared to the previous day (day 8). One reason for an increase in ACS could be that subject in 7B had a friend visiting him on day 6 and a birthday party on day 7.

The overlay of food makes a reliable prognosis difficult. In addition, there are fluctuations that may be related to special events in everyday life, which makes it difficult to identify possibly ADHD-promoting foods.

### 3.6. Anamnestic Intolerance Prior to OD and Observed Sensitivity during Food Reintroduction

In an initial anamnesis, the children were asked about their eating habits. In addition to “likes” and “dislikes,” they were also asked about suspected sensitivity as a possible trigger of ADHD. Of the 16 subjects, eleven (68.8%) did not match the information in the anamnesis questionnaire with the sensitivities found later. Of the 16 subjects, there is a match between a favorite food (eggs) and a sensitivity only in one participant (6.25%). One presented (6.25%) a correspondence of a dislike (fish) with an incompatible food. In three of the 16 subjects (18.75%) the suspicion was confirmed. The questionable foods are milk (2) and soy (1). In all three participants, physical symptoms in addition to ADHD symptoms occurred after consuming these foods.

## 4. Discussion

A main aspect of the study is to show that OD is a useful method to find out if there are food related changes of ADHD symptoms in children. Furthermore, our study is the first to investigate the individual behavioral responses on different foods related to ADHD symptoms.

Pelsser recommends that a change in diet should be considered in all children with ADHD, as Pelsser’s study showed a significant effect in children with ADHD and ODD of the elimination diet. This requires medical supervision and parental cooperation when following this restrictive elimination diet [14]. In a previous study we could also show the positive effects of OD in children with ADHD [22].

Before starting the diet, it is not obvious if children react to food, and when they do so, what kind of foodstuff result in an increase of ADHD symptoms. Children included in this study did react after 4 weeks of diet. The participants showed individual food sensitivity concerning type of food, intensity or pattern of reactivity. In all patients, ADHD symptoms were intensified by various foods during the food challenge after OD.

After detecting food related ADHD symptoms by OD, it is important to find out, by reintroduction, to what kind of food the children are sensitive. In our small sample we could demonstrate 27 different foods which increased the symptoms of ADHD in our participants.

Comparing this study with previous studies on the OD by Egger et al., a concordance of frequency of intolerance more often regarding milk products can be shown. Cow’s milk sensitivity occurred in 64% of the cases in Egger and Carter, in 60% of the cases in Hiedl, and in our current study in 68% of the cases. From this, one can conclude that the most common food intolerance that leads to an increase in ADHD symptoms is cow’s milk. However, not all of the food sensitive children did react to milk, so there should never be a general recommendation to avoid milk in the context of ADHD. In all four studies, wheat and grains in general could be detected as common provoking foods. Egg was also more often on the list of intolerant foods in all studies. [11,13,14,16,37,38], but only individual dietary recommendations should be given in the context of the individually detected food sensitivity.

The study also shows that almost every participant experienced an increase of far more than two points in ACS after the food challenge. The amount and type of reaction were individually different. The strongest reaction was seen in one subject after taking paprika. There was an immediate increase in ADHD symptoms by 25 points as measured by ACS.

It could not be shown that a connection between certain foods and behavior can already be established during the pre-diet phase by evaluating the food and behavior diary. The pre-diet phase should, however, continue to be at the forefront when carrying out an OD to observe usual eating habits and to train the protocoling. It is also useful to have a pre-diet control protocol in order to test whether the change in daily life when focusing on nutrition and behavior of the child does influence ADHD symptoms or not.

An OD with subsequent reintroduction can be the most useful diagnostic tool to identify individual food sensitivity in connection with ADHD. Because every participant reacted very individually to different foods, there must be an individual dietary recommendations for each individual child.

Several possible different pathomechanisms are noted to cause an increase in symptoms. Most children (70%) reacted to milk products. Of these, 73% did not react to dairy products which were free of lactose. This leads to a hypothesis that the pathomechanism of lactose intolerance could be connected to ADHD symptoms. In a previous study from Edreffy et al. [39], the differences in oligosaccharide metabolism between ADHD and healthy controls were described. This study supports the hypothesis that carbohydrate metabolism differs in ADHD subjects compared to control. Alabaf et al. [40] investigated physical health in children with a neurodevelopmental disorder on the basis of the database of The Children and Adolescent Twin Study in Sweden (CATSS). Their results showed that children 9–12 years of age diagnosed with ADHD suffered more than twice as often from lactose intolerance as the age-matched total population. The occurrence of celiac disease was also described to be higher in patients with ADHD. The prevalence of celiac disease increased significantly in patients with ADHD and comorbidity such as autism spectrum disorder and learning disorder.

We found ADHD related symptoms worsening in children after the consumption of different grains. This could indicate probable digestive problems in the context of different grains. Niederhofer et al. [41] reported an overrepresentation of celiac disease, identified by measuring the celiac specific antibodies anti-gliadine and anti-endomysium in patients with ADHD. Ten out of 67 patients with ADHD were diagnosed with celiac disease. A significant improvement of ADHD symptoms under gluten-free diet was observed by patients and parents. In our study, we also found remission of ADHD symptoms in grain sensitive children after three days of grain-elimination during the food reintroduction.

“Brain-gut axis” and “microbiome” have also been shown to be related to mental disorders [42,43]. Though the microbiome in humans shows a high interpersonal variation, its composition is influenced by geography, culture, and diet [44,45]. The type of nutrition plays a decisive role in the composition of the microbiome. Despite the evidence that abnormal development of the intestinal microbiome has long-term implications on host health, the causal contributions of abnormal intestinal microbiome variations to disease states have yet to be elucidated [45].

One can therefore consider whether there is a complicated mechanism behind ADHD symptoms in relation to food intake. The microbiome can be regarded as a trigger or amplifier of ADHD symptoms [43]. Kumperscak et al. found improvements in children´s behavior after three months probiotic treatment with “*Lactobacillus rhamnosus*” [46]. A randomized controlled trial published in 2016 showed a significant improvement in ADHD symptoms in children with autism after supplementation of “Lactobacillus Plantarum PS128” [47]. These results reinforce the hypothesis of microbial influence on ADHD symptoms.

Pelsser investigated the immunological response comparing allergic and non-allergic reactions in food intolerances and food allergies in children with ADHD. IgE is implicated in typical food allergies. If reactions to foods are not mediated by IgE, the assessment of IgG levels might be useful, when considering the aim of establishing a relation between foods and ADHD. According to this theory, eating foods that induce high IgG levels would lead to a substantial behavioral relapse whereas eating foodstuff that induces low IgG levels would not. The results did not support this hypothesis [14].

This suggests that food sensitivities in ADHD are not necessarily allergic reactions. However, a cell-mediated allergic reaction has not been investigated and therefore cannot be excluded. A pilot study from Dieterich et al. [48] could identify inflammatory processes in the gut reflected in inflammation-related intolerance reactions to foods, without showing a systemic inflammation in blood parameters. Food intolerances relate to high interferon IFN-γ concentrations in different gut regions. Gut mediated reactions to food intolerances showed differences to inflammation parameters in food allergy. In different gut regions, they found an increase of IFN-γ which might result from an unspecific immune response to an intestinal dysbiosis in the intestine and to a release of micro-biotic peptides as described by Farin et al. [49]. This might explain our results concerning the individual reactions to intolerant foods in food reintroduction. We found different intensities of reactions and individual time-courses to recovery. This might be related to a highly individual gut microbiome and subsequently its metabolites, which can stimulate local inflammatory processes in the gut.

This supports the theory that the composition of gut microbiome plays an important role in this context.

Whatever mechanisms a dietary intervention relies on, Stevenson et al. [50] pointed out in their research review form 2014 that a restricted elimination diet might be beneficial for ADHD symptoms in children and adults.

### Limitations and Future Directions

The evaluation of the daily behavior could, besides dietary intervention, also be impacted by various factors such as social interactions or physical health.

For the assessment of ADHD symptoms, they must occur in at least two contexts (usually at home and at school). Unfortunately, teacher´s ratings could not be collected in total. This is a clear limitation of the study.

The study was open, non-randomized, without a control group and without blinding the diet. Focusing on behavior and eating habits while implementing serious changes in daily life might lead to a remarkable bias in parent’s ratings. To corroborate these preliminary findings, an extension of the study with a larger number of subjects would be important to confirm the effects already observed.

Children had to control their eating behavior by themselves. Whether children always provided their information on food consumption truthfully is not guaranteed. Although the importance of correct information for the diary was clearly explained, there certainly were undocumented dietary violations.

## 5. Conclusions

The European treatment guidelines on ADHD recommend that restricted elimination diets may be beneficial for children with ADHD and with a history of adverse reactions to food. However, our data indicate that there are many ADHD children without a history of adverse reaction to food that may profit from OD and subsequent identification of highly individual food sensitivity.

In summary of the available results, Oligoantigenic Diet seems to be a useful tool to identify food sensitive ADHD patients. Subsequently, detected individual food sensitivities leading to individualized dietary recommendations are useful as an additional option to the existing multimodal therapy concept.

## Figures and Tables

**Table 1:** Participants’ characteristics.

Included (*n*)	16 out of 28
Age (means ± SD (range))	9.25 ± 1.73 (7–13)
Gender (m/f)	13/3
Subtypes c/hi/i (*n* = 28) Responder (*n* = 16)	16/10/2 9//6/1
Comorbidity	Dyslexia (F81.0, *n* = 6) Dyscalculia (F81.2, *n* = 2) Oppositional Defiant Disorder (F91.3, F91.8, *n* = 2) Autism (F84.0, *n* = 2) diagnosed in the course of the study Encopresis (F98.1, *n* = 1)

Subtypes: c: combined type; hi: predominantly hyperactive/impulsive; i: predominantly inattentive.

**Table 2:** Characteristics of dropped out participants.

Dropped out (*n*)	12 out of 28
Age (means ± SD (range))	10.5 ± 1.86 (8–14)
Gender (m/f)	9/3

**Table 3:** Means and standard deviations of behavioral reactions to dairy products of Group 1 (*n =* 5).

Day	M	SD	*t*(4)	*p*
dE	−3.40	0.89	−8.50	0.001
dE + 1	1.40	2.30	1.36	0.246
dE + 2	3.60	4.83	1.67	0.171
dE + 3	2.00	6.16	0.73	0.508

**Table 4:** Means and standard deviations of intolerance reactions to dairy products of Group 2 (*n* = 6).

Day	M	SD	*t*(5)	*p*
dE	8.17	4.36	4.59	0.006
dE + 1	5.83	2.32	6.17	0.002
dE + 2	4.17	5.74	1.78	0.136
dE + 3	7.67	7.34	2.56	0.051

**Table 5:** Means and standard deviations of intolerance reactions to corn of Group 1 (*n* = 2).

Day	M	SD
dE	−2.0	0
dE + 1	11.0	5.66
dE + 2	5.5	6.36
dE + 3	0	2.83

**Table 6:** Means and standard deviations of behavioral reactions to corn of Group 2 (*n* = 5).

Day	M	SD	*t*(4)	*p*
dE	4.40	4.83	2.04	0.111
dE + 1	7.80	3.42	5.10	0.007
dE + 2	6.80	5.40	2.81	0.048
dE + 3	7.40	8.68	1.91	0.129

**Table 7:** Means and standard deviations of behavioral reactions to grain (*n* = 6).

Day	M	SD	*t*(5)	*p*
dE	4.83	4.67	2.54	0.052
dE + 1	3.17	2.14	3.63	0.015
dE + 2	2.33	1.63	3.50	0.017
dE + 3	1.00	2.10	1.17	0.296

## Data Availability

The data are not publicly available according to description of confidentiality and data sharing procedures described in the study’s informed consent and assent documents.
